# Understanding support needs for family-participatory prehabilitation before hepatectomy: a qualitative study on Chinese patients and family caregivers

**DOI:** 10.3389/fpubh.2026.1916113

**Published:** 2026-09-03

**Authors:** Chuan You, Huan Li, Norshafarina Shari, Jingdong Li

**Affiliations:** 1 Department of Hepatobiliary Surgery, Affiliated Hospital of North Sichuan Medical College, Nanchong, Sichuan, China; 2 School of Graduate Studies, Management and Science University, Shah Alam, Selangor, Malaysia; 3 Sichuan Clinical Research Center for Digestive Diseases, Nanchong, Sichuan, China

**Keywords:** dyadic qualitative research, family caregiver, hepatectomy, implementation, liver cancer, patient autonomy, prehabilitation

## Abstract

**Background/objectives:**

Prehabilitation can improve patients’ physical and psychological reserve before hepatectomy, but its value depends on implementation in daily life. Because prehabilitation mainly takes place at home, family caregivers may strongly influence whether patients can follow the program. It remains unclear how family caregivers can be involved effectively. This study explored the support needs, barriers, and facilitators experienced by patients awaiting hepatectomy and their family caregivers, with the aim of informing a family-participatory prehabilitation program that fits real clinical needs.

**Methods:**

A qualitative descriptive dyadic study was conducted at a tertiary hospital in Sichuan Province, China. Purposive maximum variation sampling was used to recruit participants. Guided by a semi-structured interview outline, trained clinical nurse researchers separately interviewed 11 patient-caregiver dyads from February to June 2026. Two researchers independently coded the verified transcripts, and the wider team developed themes through discussion.

**Results:**

The patients included nine men and two women aged 43–73 years (mean, 57.4 years). Most family caregivers were patients’ spouses (*n* = 8). Six themes emerged: professional authority helped dyads accept prehabilitation; disease status, finances, work, and family roles shaped preoperative uncertainty; families acted as the main implementation unit; feasibility was limited by symptoms, knowledge gaps, family relationships, and available resources; family support was not always facilitative when patients felt their autonomy was reduced; and participants preferred support that was specific, personalized, low-burden, and sustainable. Patients and caregivers often described burden and acceptable supervision differently.

**Conclusion:**

Family involvement should not be reduced to asking relatives to monitor patients. A workable family-participatory model needs professional guidance, negotiated patient-caregiver roles, protection of patient autonomy, assessment of caregiver capacity, low-literacy delivery, and attention to cost. These findings provide a practical basis for developing family-participatory prehabilitation interventions before hepatectomy.

## 1 Background

Primary liver cancer is still a major cause of cancer death worldwide. China has a large share of these cases (1). For patients whose liver cancer can be removed, hepatectomy is still the main curative treatment. However, recovery can be difficult. Patients may have limited liver reserve, poor nutrition, stress, or postoperative complications (2). The time before surgery is therefore important. It can be used to help patients become stronger before treatment, but this chance is often not fully used.

Prehabilitation is given before main treatment. Its aim is to improve physical and psychological reserve. It often includes exercise, better nutrition, changes in unhealthy habits, and psychological support (3–7). For patients waiting for hepatectomy, preoperative exercise can improve heart and lung function (3). Studies in major abdominal surgery also show possible benefits for recovery and postoperative outcomes (4, 5). Unlike other major abdominal surgeries, hepatectomy carries a unique risk of postoperative liver failure due to limited hepatic reserve and underlying chronic liver disease, making preoperative functional optimization particularly critical. However, success in trials does not always mean easy use in daily care. Patients must understand the advice. They must adjust it to their symptoms and available resources. They also need to keep doing it during a short and uncertain preoperative period.

In real life, patients rarely do this alone. Family caregivers often prepare meals. They help with daily activities. They explain medical information. They arrange transport and money. They also respond to patients’ emotional distress. Family-centered cancer interventions can help both patients and caregivers (8). However, caregivers often have unmet needs. These needs may be informational, emotional, or practical (9, 10). If families are seen only as extra workers, clinical tasks may simply be moved into the home. Caregiver capacity and family relationships may then be overlooked.

This problem may make family-involved care ineffective. Close family involvement may bring continuity, familiarity, and motivation, and it can also lead to over-monitoring, conflict, hidden emotions, or pressure to follow advice. Patients and caregivers may see the same situation differently. Some caregivers may think repeated reminders are necessary, while some patients may not like to be restricted. Some caregivers may see supervision as protection, but some patients may feel it as a loss of control. A dyadic design keeps these different views visible. It does not force them into one single account.

In summary, for patients awaiting hepatectomy, prehabilitation is typically carried out at home during a short and uncertain preoperative window, making family caregivers the de facto implementers. Yet most prehabilitation studies have focused solely on clinical outcomes and patient adherence, leaving a critical gap: how can family caregivers be involved effectively and safely, without undermining patient autonomy or overburdening the family. A dyadic, qualitative exploration is therefore essential to ground the development of a culturally appropriate, family-participatory prehabilitation program for this specific population.

This study therefore asked three questions: (1) How do patient-caregiver dyads understand and evaluate family-participatory prehabilitation? (2) What needs, barriers, and facilitators affect implementation? (3) How can family roles support adherence without reducing patient autonomy or placing too much burden on caregivers?

## 2 Methods

### 2.1 Study design

We used a qualitative descriptive design (11, 12). We also used a dyadic analytic orientation. This design helped us stay close to what participants said. It also helped us produce findings that may guide future clinical intervention development. In addition, it allowed us to compare views within each patient-caregiver dyad and across different dyads. We reported the study according to the Consolidated Criteria for Reporting Qualitative Research (COREQ) (13).

### 2.2 Setting, participants, and recruitment

The study was carried out in the Department of Hepatobiliary Surgery. The hospital was a tertiary hospital in Nanchong City, Sichuan Province, China. The ward nurses were responsible for recruiting the patient-caregiver dyads. The sample included 22 adults forming 11 patient-caregiver dyads. Each dyad consisted of one patient awaiting hepatectomy for hepatocellular carcinoma and one main family caregiver. Interviews were conducted from February 8 to June 5, 2026.

Patients had to meet these criteria: age 18 years or older; confirmed hepatocellular carcinoma; planned elective hepatectomy; expected waiting time before surgery of at least 2 weeks; clear consciousness; ability to speak and communicate; family members providing basic daily care; and voluntary written informed consent. Patients were excluded if they had cognitive impairment, severe mental disorders, or physical illness that made interview participation difficult. They were also excluded if they had no main family caregiver who could provide support, or if they refused to take part.

We used purposive sampling with maximum variation. This helped us recruit patient-caregiver dyads with rich information. Sampling considered patient age, sex, education, residence, disease status, surgical history, preoperative waiting time, and family composition. It also considered the caregiver’s relationship with the patient, caregiving experience, cohabitation, employment, and level of involvement. Patients and caregivers were interviewed separately. Data collection and analysis were carried out at the same time. Recruitment stopped when later interviews added depth to existing themes but did not add many new ideas. The sample had sufficient information power because it combined a focused study aim, a specific but maximally varied sample, and separate semi-structured interviews conducted by trained nurses. The focused research questions, rather than a single substantive theory, provided the analytic structure, while established thematic and framework methods, independent coding, and within- and across-dyad matrices supported systematic analysis (13–15).

### 2.3 Data collection

Two researchers interviewed patients and caregivers separately. All interviews were held in private physician-patient communication rooms in the hepatobiliary surgery ward. The rooms were quiet and closed. This helped protect privacy and reduce disturbance.

The interviews followed a semi-structured guide. Questions focused on how participants understood prehabilitation, what benefits they expected, and what worries they had before surgery. We also asked about exercise, nutrition, family task sharing, barriers, preferred health education methods, and suggestions for family-participatory prehabilitation programs.

A total of 22 interviews were completed. Interview length ranged from 14.6 to 36.1 min. The mean length was 22.7 min, and the median length was 21.6 min. The total interview time was about 499 min.

The principal investigator led the interviews. Another research team member assisted. Both were clinical nurses trained in qualitative interviewing. With participants’ consent, all interviews were audio-recorded. During the interviews, the second researcher took field notes on facial expressions, gestures, and other non-verbal cues.

All interviews were conducted in Mandarin, with some Sichuan dialect. Recordings were transcribed word for word. The research team checked each transcript against the recording sentence by sentence. Participants then reviewed the final transcripts to confirm the content and wording.

To protect privacy, all personally identifiable information was removed before analysis. Patient codes ranged from P01 to P11. Family caregiver codes ranged from C01 to C11. The first author (CY), a member of the research team who was familiar with the study context and had demonstrated English proficiency (College English Test Band 6 and Malaysian University English Test Band 4), translated the interview quotations from Chinese into English. The translation prioritized conceptual equivalence and preservation of the original tone and meaning. All translated quotations were reviewed against the original Chinese transcripts by the research team to ensure accuracy and fidelity to the source data.

### 2.4 Data analysis

Data were analyzed within the qualitative descriptive design. Braun and Clarke’s six-phase reflexive thematic analysis (16) guided theme development, while the framework method (15) was used to organize coded data and case summaries into matrices. These approaches formed a single sequential process: the research questions provided the initial focus, inductive coding allowed new themes to emerge, thematic analysis guided coding and theme development, and framework matrices supported systematic dyadic comparison.

Two researchers repeatedly read the verified transcripts many times. They coded the data independently. The analysis followed several steps. We first became familiar with the data. We then developed initial codes, summarized each case, grouped related codes, refined themes, and selected representative quotes.

The individual transcript was the initial coding unit, and the patient-caregiver dyad was the relational unit of analysis. Patient and caregiver codes and case summaries from each matched pair were organized in a dyadic matrix. We compared the two accounts within each dyad to identify shared and divergent views, while considering family context and practical constraints. We then compared the matrices across all 11 dyads to identify common patterns and contrasting cases.

After independent coding, the two coders compared their interpretations. They discussed differences and refined the evolving analytic framework together. The full research team then reviewed the codes, dyadic matrices, candidate themes, and supporting quotations.

Six themes were retained: acceptance, uncertainty, family structure, feasibility barriers, autonomy, and intervention preferences. Theme frequency was used only to show how widely a theme appeared across cases. It was not treated as statistical frequency or effect size.

To enhance the credibility of the study, we implemented several measures, including sentence-by-sentence verification of audio recordings against transcribed texts, independent coding by two researchers, participant confirmation of interview transcripts, comparison of consistent and inconsistent statements in paired cases, careful examination of counterexamples, and collective review of the final thematic framework by the team (17). Following theme identification, we developed a thematic relationship map to illustrate the intrinsic connections between professional legitimacy, uncertainty, family implementation, feasibility barriers, autonomy, and sustainable support.

### 2.5 Ethical considerations

The study was approved by the Ethics Committee of the Affiliated Hospital of North Sichuan Medical College on 9 February 2026 (approval no. 2026ER91-1). All participants provided written informed consent. The study was conducted in accordance with the Declaration of Helsinki (18).

## 3 Results

### 3.1 Participant characteristics

**Figure 1:**
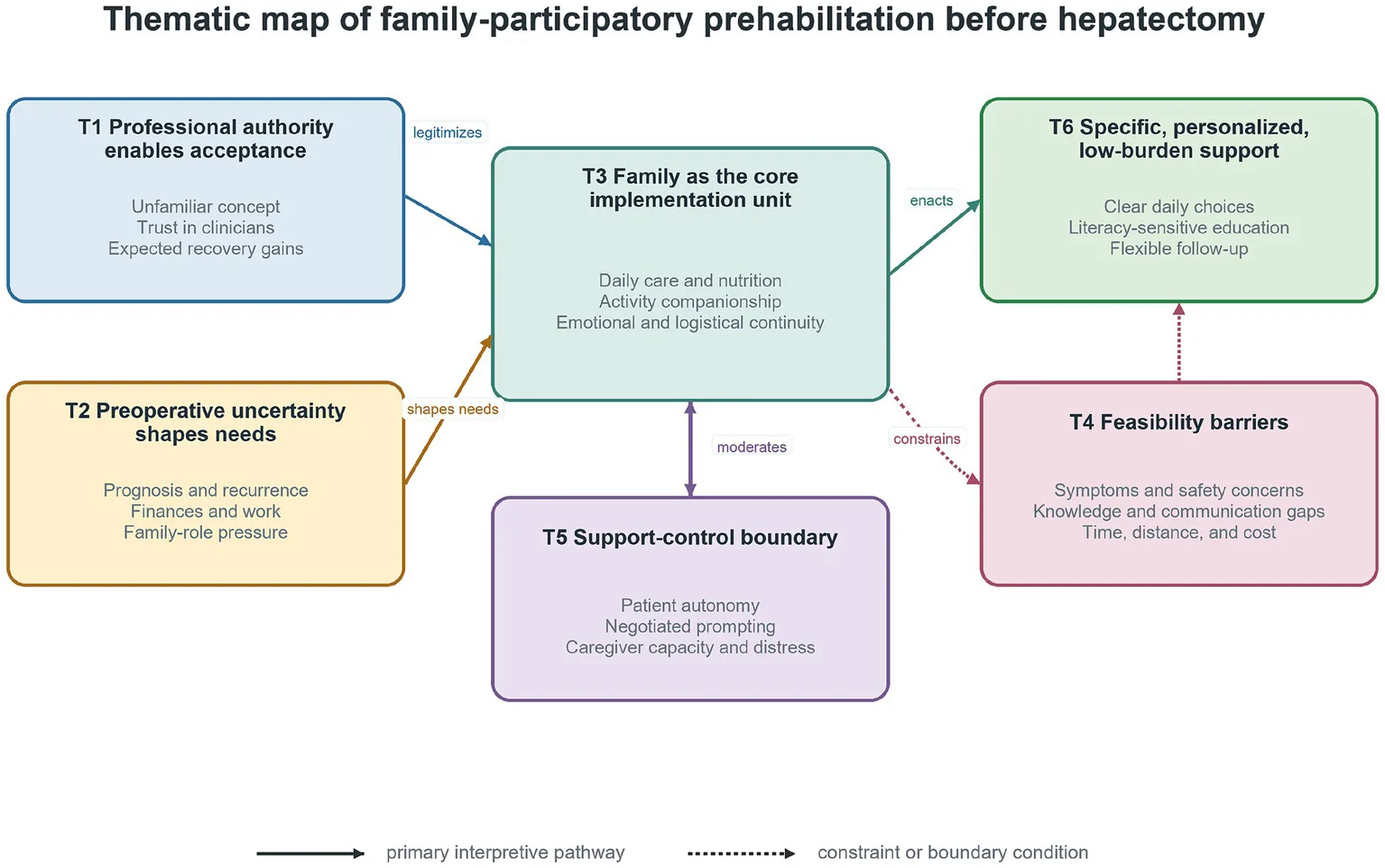
Thematic map of family-participatory prehabilitation before hepatectomy. This map summarizes the six interrelated themes derived from the qualitative analysis: Professional authority is conducive to patients’ acceptance of prehabilitation treatment; Preoperative uncertainty determines the support needs of patients and family caregivers; The family, as the core implementing entity, converts prehabilitation measures into continuous daily care, activity companionship, as well as emotional and logistical support; Feasibility obstacles and the support-control boundary limit the implementation effect, indicating the need to provide specific, personalized and low-burden support plans to safeguard patients’ autonomy and caregivers’ capabilities; Solid arrows represent the main explanatory paths, while dashed arrows indicate constraints or boundary limitations.

The study included 11 patient-caregiver dyads (22 participants). Nine patients were men and two were women; ages ranged from 43 to 73 years (mean 57.4 years). Patient education was generally limited: one had no formal literacy, three had primary education, six had junior-high education, and one had senior-high education. Caregivers were eight spouses, two adult sons, and one brother. Six caregivers had primary education and five had junior-high education. The corrected baseline information for Dyad 11 identified a 45-year-old male patient and his 43-year-old wife; both had junior-high education, the household reported an annual income below CNY 100,000, one daughter, and no previous caregiving experience (Tables 1, 2, Figure 1).

**Table 1:** Participant characteristics.

Dyad	Patient sex, age	Patient education	Patient occupation	Caregiver relationship; sex, age	Caregiver education	Caregiver occupation
01	M, 58	Junior high	Worker	Wife; F, 55	Primary	Worker
02	M, 44	Junior high	Decorator	Wife; F, 40	Junior high	Not employed
03	M, 53	Junior high	Not employed	Wife; F, 52	Primary	Not employed
04	F, 56	No formal literacy	Farmer	Husband; M, 58	Junior high	Construction
05	M, 43	Senior high	Sales	Wife; F, 44	Primary	Retail
06	M, 73	Primary	Retired/resident	Son; M, 48	Junior high	Worker
07	M, 69	Junior high	Retired/resident	Wife; F, 61	Primary	Retired/resident
08	M, 69	Primary	Farmer	Brother; M, 62	Primary	Retired
09	F, 53	Junior high	Self-employed	Husband; M, 52	Primary	Self-employed
10	M, 68	Primary	Not employed	Son; M, 40	Junior high	Worker
11	M, 45	Junior high	Sales	Wife; F, 43	Junior high	Sales clerk

**Table 2:** Themes, subthemes, and illustrative quotations.

Theme	Interpretive theme	Key subthemes	Illustrative quotation(s)
1	From unfamiliarity to acceptance: professional authority helped liver cancer patients and family caregivers accept prehabilitation.	Cognitive gaps or incompleteness; trust in the clinicians; expectation of a faster recovery	“I had never heard of this.” (P03) “Anyway, I’ll just follow the doctor’s instructions.” (C01)
2	Disease status, economic situation, and family roles can all give rise to preoperative uncertainty among patients and their family caregivers.	Prognostic uncertainty; recurrence; work and financial pressure; concealed distress	“Every month, I gotta pay off my mortgage and cover my kid’s tuition” (P11)
3	Family was the core implementation unit	Food preparation; activity companionship; emotional soothing; continuity between the hospital and the home	“After the surgery, he felt a lot more at ease when his family stayed by his side.” (C11)
4	Feasibility is mainly related to physical conditions, knowledge level, interpersonal relationships and access to resources.	Poor appetite; pain and fatigue; vague nutrition/exercise knowledge; conflict; time and cost constraints	“You asked them to work out, but they did not really cooperate.” (C05)
5	Family support is not always a facilitating factor.	Disagreement between patients and caregivers; concern about burdening relatives; resistance to monitoring; caregiver psychological distress	“Do not force me.” (P11) “My mental state is not very good either.” (C06)
6	Participants wanted specific, personalized, low-burden, sustainable support.	Daily choices; clear exercise and light-diet guidance; multimodal health education matched to literacy level; flexible follow-up methods	“Could the doctor give us some kind of checklist?” (C11)

### 3.2 Theme 1: from unfamiliarity to initial acceptance: trusted professional guidance facilitated engagement after explanation

Most participants did not have a clear idea of prehabilitation before the interview. They usually understood preoperative preparation as rest, intravenous fluids, light food, or simply following hospital advice.

After the interviewers explained the prehabilitation treatment, most participants expressed their acceptance. The acceptance was mainly due to their trust in the doctors and their expectation of clear therapeutic effects. These effects included being able to get out of bed earlier, being discharged from the hospital faster, and returning to work earlier.

*“I’ve never heard of anything like this.” (P03)*

*“Anyway, I’ll just follow the doctor’s instructions.” (C01)*

Therefore, the professional advice of the doctor can quickly make the unfamiliar prehabilitation plan acceptable. However, it should be noted that when the advice is too vague, it is difficult for the patient’s family to convert the macroscopic goals into daily actions. Participants need specific guidance on the exercise dosage, types of diet and intake amounts. It is also need to note that since the acceptance attitude was elicited during the interview process, it should not be interpreted as evidence of consistent compliance.

### 3.3 Theme 2: disease status, economic situation, and family roles can all give rise to preoperative uncertainty among patients and their family caregivers

Some people might initially say, “I’m not worried at all,” but after further conversation, it becomes clear that they actually have many concerns. What does the diagnosis mean? How severe is the condition? Will it recur? What is the prognosis? How much does the surgery cost? How will it affect their work? How should they handle the care of their older family members and children? These questions continue to cause varying degrees of distress for them. Some deliberately maintain an optimistic attitude; some view it as an inevitable fact; and some completely avoid mentioning it - each person has their own coping strategy. But it is certain that a seemingly non-anxious state does not equate to psychological calmness.

*“What exactly is this illness? Is it cancer?” (P08)*

*“I’m afraid of recurrence and spread… it’s a burden on my family.” (P09)*

For some patients, the rehabilitation process not only involves physical recovery but also requires dealing with work stress, mortgage repayments, and the responsibility of supporting the family. P11 states that due to concerns about work, monthly payments, and children’s tuition fees, they cannot sleep at night and feel exhausted during the day. Caregivers also face many challenges: they must balance their own work and caregiving responsibilities, frequently accompany the patients to medical appointments, handle household chores, and deal with uncertain future family prospects.

*"Every month, I gotta pay off my mortgage and cover my kid's tuition" (P11)*

*“Constantly going back and forth, I simply can’t fall asleep.” (P11)*

Although these concerns were not unique to prehabilitation, they affected its uptake and implementation. Uncertainty about the disease and surgery could reduce the priority given to an intervention whose benefits were not immediately visible. Financial, work, and family responsibilities also limited the time, emotional energy, and caregiver availability needed for education, meal preparation, exercise accompaniment, and follow-up. We therefore interpreted these concerns as contextual factors affecting patients’ and caregivers’ readiness and capacity to engage in prehabilitation.

### 3.4 Theme 3: family was the core implementation unit

Family members undertook most of the assistance work during the patient’s prehabilitation stage. This included tasks such as cooking, assisting with daily activities, arranging transportation, communicating with the hospital, providing financial support, and offering emotional comfort. Respondents generally believed that compared to hired caregivers, family members were more familiar with the patient and could provide assistance at any time, bringing them a stronger sense of security. Therefore, family participation is not an optional supplementary measure but a key element for ensuring the effective implementation of prehabilitation outside the hospital environment.

*“After the surgery, he felt a lot more at ease when his family stayed by his side.” (C11)*

However, the participants also clearly stated that although family members can carry out and comply with these tasks, risk assessment, treatment plan formulation, method guidance, and deviation correction still fall within the scope of responsibilities of healthcare professionals. They hope that healthcare professionals can provide reliable prehabilitation guidance, while family members can implement corresponding measures flexibly based on the actual situation.

*“I prefer using what’s available at home.” (P03)*

### 3.5 Theme 4: feasibility is mainly related to physical conditions, knowledge level, interpersonal relationships and access to resources

There are also several factors that affect the degree to which patients follow the prehabilitation plan, and they do not solely depend on personal will. For instance, physiological challenges such as loss of appetite, pain, fatigue, and limited mobility, as well as patients’ concerns about injury during exercise, uncertainty about dietary requirements and exercise methods and intensity. Some participants regard household chores or farm work as exercise; in terms of nutrition, although the concept of “light diet” sounds simple, when it comes to actual operational tasks (such as purchasing ingredients, choosing cooking methods, calculating protein intake and food portions), they often have no idea where to start. These factors collectively increase the difficulty of implementation.

*“For us, the exact meaning of ‘a light diet’ remains quite unclear.” (C11)*

*“He can’t eat anything at all; he simply has no appetite, which makes me very anxious.” (C06)*

The physiological and informational challenges have not been fully resolved, and the relationships within the family and environmental factors have introduced new obstacles. Caregivers pointed out that patients sometimes exhibit irritable emotions, refuse to eat, and even remain unresponsive even after repeated persuasion; moreover, work schedule arrangements, commuting distances, limited income, and the burden of responsibilities towards older relatives and children all significantly weaken the caregivers’ ability and energy for care. These findings make us realize that the compliance of prehabilitation should not merely be regarded as the “non-adherence to medical advice” of the patients, but should be understood as the result of the interaction between physical conditions, interpersonal relationships, and real constraints.

*“You asked them to work out, but they didn’t really cooperate.” (C05)*

### 3.6 Theme 5: family support is not always a facilitating factor

Some patients hope to independently complete the prehabilitation plan and control the pace on their own, not wanting to be overly dominated by others and unwilling to cause inconvenience to their family members. While some caregivers are more concerned about timely reminders and information transmission to ensure that all necessary tasks are completed. This difference in needs leads to the fact that the support provided by family caregivers fails to achieve the expected results. Patients may consider these requirements insignificant and even feel oppressed by them, while caregivers often face problems such as patients not following the requirements for eating, not cooperating with treatment, emotional outbursts, and repeated persuasion, thus feeling overwhelmed.

*“If they frequently care about me, it actually harms my health.” (P09)*

*“Do not force me.” (P11)*

Therefore, from the perspectives of both patients and their family caregivers, the concept of “monitoring” may have very different meanings: caregivers view it as a protective measure, while patients consider it excessive control, especially when the monitoring behavior is overly intense. When family involvement is interpreted as strict supervision, problems such as conflicts, concealment, perfunctory responses, and even complete avoidance may follow. Of course, different people’s tolerance levels for the intensity of monitoring also vary.

Additionally, the coping abilities of caregivers are also worthy of attention; some relatives admit that seeking a balance between work responsibilities and caring for patients brings great physical and mental stress.

*“My mental state is not very good either.” (C06)*

### 3.7 Theme six: participants wanted specific, personalized, low-burden, sustainable support

Participants tend to prefer guidance plans that are highly operational and clear and concise: including daily exercise plans with clearly marked frequencies and intensities; a clear definition of what constitutes dangerous behavior and when exercise should be stopped; and how adjustments should be made for situations such as pain or fatigue that make exercise difficult. In terms of nutrition, it is hoped to provide the types of locally available foods and their reasonable combinations, as well as cooking methods.

*“Could the doctor give us some kind of checklist?” (C11)*

For the guidance methods for prehabilitation, they need to vary according to different cultural levels and age groups of patients and caregivers. Some patients and their family members prefer face-to-face demonstration teaching; others prefer to use simple and clear paper textbooks; and some people prefer to watch short videos, use WeChat or conduct follow-up communication. Given that the educational background of some patients and their family caregivers is limited and they may have difficulty proficiently using smartphones, relying solely on online teaching channels is clearly insufficient.

*“She does not recognize some of the characters; sending instructions via WeChat would be ineffective.” (C03)*

*“It’s best for medical staff to guide her step-by-step, or sending a video demonstration would also suffice.” (C11)*

Participants generally have an aversion to the high-intensity training courses and the daily check-in reports. They hope for at least a 1-week introductory transition period. During this period, simple and clearly-defined content should be adopted, as this is more acceptable and easier to follow. It is worth noting that patients still wish to independently choose their training time and intensity.

*“Maybe start one week in advance, giving us a little bit each day.” (C11)*

*“I can do it according to my own time; I do not need someone supervising me.” (P09)*

## 4 Discussion

### 4.1 Principal findings

This study, as an important part of developing a family-participatory prehabilitation program, focused on conducting dyadic interviews to explore the support needs, obstacles, and facilitating factors during the family-participatory prehabilitation process. The analysis results indicated that the family is not only a place for implementing prehabilitation treatment but could also be a source of burden or control factors. Therefore, family participation should not be regarded as an automatic beneficial outcome. Its effectiveness depends on the way professional personnel provide guidance, whether the patient’s autonomy is guaranteed, whether the caregivers have sufficient capabilities, and whether the resources available in the family are feasible.

By further analyzing the six core themes that emerged from this study, it was found that the recognition of professional institutions could motivate patients to take the first step; the uncertainty before the surgery determined the specific needs for support of liver cancer patients and their family caregivers; and the family plays a crucial role throughout the process, helping patients convert the prehabilitation plan into action. However, the feasibility of this method depends on the following factors: whether the caregivers have sufficient capabilities to provide care, whether the boundary between support and control is clearly defined, and whether it is further limited by factors such as the severity of symptoms, knowledge level, communication quality, availability of time and resources, travel distance, and economic cost. Ultimately, improving the compliance of liver cancer patients with prehabilitation not only requires the presence of family members but also requires finding a balance point that is acceptable to both parties in terms of professional guidance, care capabilities, patient autonomy, and intervention burden.

### 4.2 The authoritative guidance from professional medical staff is definitely a necessary condition for initiating prehabilitation, but it is not a sufficient one

Most patients with liver cancer and their family caregivers were largely unaware of prehabilitation at the beginning, but they still agreed to accept the program after a brief introduction by the medical staff. This indicates their trust and reliance on medical authorities, and also explains why the trust in doctors during this short period before the surgery can significantly alleviate patients’ concerns and accelerate the recovery process. However, if the medical staff rely solely on authority without providing specific operational guidance, patients with liver cancer and their family caregivers often can only give verbal commitments that lack substantive participation. This is crucial for building family-participatory prehabilitation programs. The results observed in clinical trials and meta-analyses (3–7, 19) are based on standardized treatment procedures; in routine practice, patients and caregivers must clearly understand what operations should be performed, why these operations are performed, when to terminate treatment, and when to adjust the prehabilitation plan in order to ensure the effectiveness of the intervention measures.

This discovery is consistent with the conclusion of another qualitative study on pre-operative rehabilitation training for cancer patients: the patients’ acceptance depends on their personal perception of the prehabilitation plan, their willingness to cooperate in practice, and the degree of participation by medical staff (20). Research on medical personnel also indicates that issues such as poor coordination among stakeholders, improper scheduling, insufficient staffing, and unclear delineation of responsibilities all hinder the integration of rehabilitation training into routine medical practice (21, 22). Therefore, in guiding patients and their family caregivers through the prehabilitation process, professional guidance should not be limited to vague suggestions such as “increase physical activity” or “follow a light diet,” but should include personalized assessment and plan formulation, demonstration of operational steps, real-time adjustment of plans based on feedback, safety norms, and clear contact information in case of problems. General instructions such as “increase physical activity” or “follow a light diet” should be replaced with detailed guidance plans tailored to specific symptoms, cultural backgrounds, locally available ingredients, and the remaining days before surgery.

### 4.3 The implementation process should take into account the stress and concerns of both the patients and the family caregivers, rather than focusing solely on one party

Our research results indicate that the design of the prehabilitation program should focus on the relationship between the patient and the caregiver rather than solely on the patient themselves. Interventions involving family care can improve the treatment outcome of the patient (8), but the needs of the caregiver themselves often go unmet (9, 10). The interview results show that there are differences in the descriptions of treatment difficulty, prehabilitation compliance, emotional distress, and supervision needs by the patient and the caregiver; therefore, a purely patient-centered approach may overlook the stress of the caregiver, while a caregiver-led approach may overestimate family resources or ignore the personal preferences of the patient.

The family should be regarded as a relational implementation system. Information, emotions, tasks, and decisions are transmitted between the patient and the caregiver. They influence each other: when the patient lacks physical strength, the family caregiver may increase the frequency of reminders; when the caregiver feels stressed, their support style may become more assertive. Therefore, when conducting an assessment before the implementation of the prehabilitation program, it is best to separately ask each participant about their desired goals, existing concerns, available time, expected role, and willingness to accept guidance. Subsequently, in the clinical discussion, a feasible plan should be jointly formulated. This method can avoid simply equating “family participation” with the need for thorough preparation or comprehensive capabilities, and can truly incorporate the actual situations of both the patient and the family caregiver into the pre-intervention assessment, thereby improving the feasibility and compliance of the prehabilitation program.

### 4.4 Maintaining a proper balance between support and control can help improve patients’ compliance with the prehabilitation plan

Some interviewees indicated that the mandatory supervision by family members made them feel uncomfortable and thus developed resistance. This suggests that the patient’s autonomy directly affects treatment compliance. When family members impose prehabilitation tasks on the patient, the patient may develop resistance, either by concealing their own needs or deliberately downplaying these needs to avoid worrying their family members; conversely, if the patient has certain autonomy in treatment arrangements, task selection, and family participation, they are more likely to cooperate in implementing the prehabilitation plan.

The boundary between support and control is not only an ethical issue but also involves considerations at the behavioral practice level. Strict monitoring is not equivalent to effective assistance. The family-centered prehabilitation model should position relatives as collaborative partners, rather than merely as “supervisors” responsible for ensuring the patient’s compliance with treatment.

Although in the context of Chinese culture, emphasizing family participation is indeed regarded as a matter of course and even encouraged, cultural factors neither eliminate individual differences nor replace respect for the patient’s right to informed consent. Culturally adaptive care plans not only need to skillfully transform family relationships into supportive resources, but also must ensure that patients retain the autonomy to decide who participates in care, which information to share, and how to provide support.

Therefore, when formulating family-participatory prehabilitation implementation strategies, it is advisable to consider allowing patients to autonomously choose their preferred notification methods, providing multiple comparable alternative activity options, and establishing exclusive channels for patients and caregivers to privately express concerns, etc.

### 4.5 Different guidance methods should be provided based on the varying health literacy levels of the participants

Participants hope to obtain information through various channels, including on-site demonstrations, printed materials, videos, WeChat messages, and phone consultations, etc. Especially for those with limited literacy skills, who have difficulty recognizing characters, are not familiar with smartphone operations, and are in rural areas with inconvenient transportation, medical staff should provide more accessible ways to receive information. Although current digital health services can indeed expand coverage, if only relying on online platforms, the most needy families may be overlooked.

Therefore, in the content design process of the family-participatory prehabilitation program, the basic principles of health literacy must be strictly followed: use simple and understandable expressions; avoid presenting too much information at once; supplement with visual charts or real-time demonstrations; require participants to repeat the information to verify the understanding level; examples should be selected from local available materials; and use telephone follow-ups as an alternative, rather than relying solely on applications.

### 4.6 Implications for intervention development and evaluation

Based on the research results, we propose an intervention framework consisting of three elements. Firstly, from the perspective of healthcare providers, the purpose of prehabilitation treatment should be clearly defined together with the patient and their family caregivers, and the risk factors of the patient should be evaluated to formulate a personalized plan; Secondly, patients and caregivers can negotiate to clearly define their specific responsibilities - including who is responsible for meal preparation, accompanying exercise, symptom recording, coordinating with the hospital, and reminder methods, etc.; Thirdly, prehabilitation measures should be in line with the actual capabilities of the family. The educational content should be customized according to the patient’s literacy level, numerical skills, work schedule, travel distance, and economic conditions. These elements are consistent with the implementation research conclusions that emphasize the acceptance, coordination, and adaptation to local service conditions (20–23).

In addition, a period of one to 2 weeks should be reserved before officially initiating the prehabilitation treatment to implement the basic intervention measures, so that the patients and their families have sufficient time to familiarize themselves with and adapt to the plan.

In terms of the formulation of prehabilitation content, a concise daily activity list should be provided, supplemented by on-site demonstrations, short videos or phone reminders to consolidate the effect. The content should cover the core elements reflected in the present findings and existing prehabilitation literature: exercise training, nutritional improvement, and psychological support (3–7, 19). Each activity should clearly recommend the intensity, alternative options, warning signals to be monitored, and the timing for stopping the activity. The follow-up assessment should be concise and to the point, dealing only with the identified issues and not requiring daily submission of detailed reports.

A study released in 2025 indicates that the efficacy of prehabilitation intervention measures varies significantly depending on the specific intervention plan and assessment criteria (19). Research evidence from China also highlights the obstacles and facilitating factors encountered during the implementation of prehabilitation intervention in surgical rehabilitation (23). This study further confirms that different participants have unique needs. Therefore, future clinical trials that incorporate family participation to improve the prehabilitation compliance of liver cancer patients should not simply consider family participation as a general and undifferentiated intervention factor; specific family behaviors must be clearly defined, detailed implementation plans for different health literacy patient and caregiver groups must be explained, and the benefits brought about by these must be evaluated to determine whether the additional voluntary efforts made by family caregivers and medical staff are justified.

### 4.7 Strengths and limitations

The main advantage of this study lies in the adoption of a dyadic design, which enables a comparative analysis of the viewpoints of patients and caregivers, thereby revealing the contradictions in the interactional relationships that are often overlooked during individual interviews. Additionally, the purposeful maximum variation sampling method effectively captured the diverse characteristics of the participants in terms of age, gender, educational background, occupation, and caregiving relationship. In terms of reliability, we ensured data quality through multiple measures: including audio text verification, independent coding by two reviewers, confirmation by the participants of the interview records, and overall team review. The thematic relationship map further clarified the potential connections among various topics.

This study also has several limitations: The research was conducted only in a tertiary hospital in Nanchong City, involving a total of 11 pairs of participants. Given that most of the participants came from remote and economically disadvantaged rural areas, their overall educational level was relatively low. While it reflected the regional characteristics, it also weakened the representativeness of the participating population. The opinions of clinical doctors, rehabilitation professionals, or patients without fixed caregivers were not included in this study. Additionally, the participants who were willing to participate in such family-centered research were likely to have more harmonious family relationships than the average. It cannot be ruled out that the respondents might show higher trust in doctors or higher treatment compliance due to social expectations. Finally, since the citations were in Mandarin and Sichuan dialect, some subtle cultural differences might not have been accurately conveyed when translating into English.

## 5 Conclusion

This single-center study included 11 patient-caregiver dyads awaiting hepatectomy for liver cancer in Sichuan, China. The findings suggest that family involvement can support prehabilitation, but it can also create burden or constraints. Family members may help with meals, daily activities, emotional support, and communication with healthcare professionals. At the same time, they may face work pressure, financial strain, and emotional distress. Family support may also become problematic when it is limited to monitoring and leaves patients little room to make their own choices.

Family-participatory prehabilitation should therefore be guided by healthcare professionals and negotiated with both patients and caregivers. Programs should also fit caregivers’ capacity, education, access to digital tools, and financial circumstances. Future studies should test whether a low-cost family-participatory model can improve collaboration, treatment adherence, and patient-centered outcomes.

## Data Availability

The original contributions presented in the study are included in the article/supplementary material, further inquiries can be directed to the corresponding authors.
